# L-Serine–Incorporated Collagen Scaffolds for Modulating In Vivo Degradation Behavior

**DOI:** 10.3390/jfb16120466

**Published:** 2025-12-18

**Authors:** Su-Young Kim, Ji-Hyeon Oh, Min-Ho Hong, Joon Ha Lee, You-Young Jo, Seong-Gon Kim

**Affiliations:** 1 Department of Oral and Maxillofacial Surgery, College of Dentistry, Gangneung-Wonju National University, 7 Jukheon-gil, Gangneung-si 25457, Gangwon-do, Republic of Korea; fmduddl20@gmail.com (S.-Y.K.); oms@gwnu.ac.kr (J.-H.O.); 2 Department of Dental Biomaterials, Research Institute of Oral Science, College of Dentistry, Gangneung-Wonju National University, 7 Jukheon-gil, Gangneung-si 25457, Gangwon-do, Republic of Korea; mhong@gwnu.ac.kr; 3 Industrial Entomology Division, National Institute of Agricultural Science, Rural Development Administration, Wanju 55365, Jeonbuk-do, Republic of Korea; coover@korea.kr (J.H.L.); yyjo@korea.kr (Y.-Y.J.)

**Keywords:** collagen scaffold, L-serine, biodegradation, silk protein, amino acid modification, bone regeneration

## Abstract

Collagen-based biomaterials are widely used, but their relatively rapid biodegradation can limit functional duration. Such collagen constructs are widely used as barrier membranes in guided tissue and bone regeneration, where controlled degradation is essential for maintaining function. Although conventional crosslinking methods extend stability, they may introduce cytotoxicity, alter mechanical behavior, or hinder tissue integration. This study evaluated whether incorporating L-serine, a polar amino acid capable of hydrogen bonding, could modulate collagen structure and slow degradation without chemical crosslinking. L-Serine was selected because its hydroxyl-containing side chain can engage in biocompatible, hydrogen-bond–mediated interactions that offer a mild, non-crosslinking means of stabilizing collagen. Collagen scaffolds, prepared by incorporating L-serine into a collagen hydrogel followed by drying, were produced with 0–40 wt% L-serine and characterized using X-ray diffraction, Fourier-transform infrared spectroscopy, circular dichroism, and scanning electron microscopy. In vivo degradation was assessed in a subcutaneous mouse model comparing unmodified collagen, collagen containing 40 wt% L-serine, and a commercially available bilayer porcine collagen membrane (Bio-Gide^®^, composed of type I and III collagen), with residual area quantified by serial sonography and histological evaluation. Low-to-moderate L-serine incorporation preserved triple-helical features, while 40 wt% led to crystalline domain formation and β-sheet enrichment. L-serine–treated collagen exhibited significantly greater residual area (2.70 ± 1.45 mm^2^) than unmodified collagen (0.37 ± 0.22 mm^2^, *p* < 0.05), although Bio-Gide^®^ remained the most persistent (5.64 ± 2.76 mm^2^). These findings demonstrate that L-serine incorporation can modulate collagen structure and degradation kinetics through a simple, aqueous, and non-crosslinking approach. The results provide preliminary feasibility data supporting amino acid–assisted tuning of collagen resorption properties and justify further evaluation using membrane-specific fabrication and application-relevant testing.

## 1. Introduction

Collagen is one of the most widely utilized natural polymers in biomedical applications due to its biocompatibility, intrinsic cell-interactive motifs, and capacity to integrate with surrounding tissues [1,2,3]. In the dental and craniofacial fields, collagen membranes are commonly applied as barrier materials in guided tissue and bone regeneration procedures, where controlled degradation is essential to maintain structural integrity and avoid premature loss of function [4,5]. Beyond barrier applications, collagen also serves as a carrier for cells, growth factors, and bioactive compounds, in which its degradation behavior directly influences mechanical stability and release characteristics [6,7].

Despite these advantages, collagen-based devices often exhibit relatively rapid in vivo biodegradation caused by enzymatic cleavage, hydrolytic processes, and cellular remodeling [8,9,10]. Such rapid resorption may compromise intended barrier duration or sustained-release functionality, particularly when residence time must be matched to biological healing phases [11]. Therefore, strategies to fine-tune collagen degradation, while maintaining biocompatibility, flexibility, and surgical handling, remain of practical interest for various biomedical uses.

Multiple approaches have been investigated to modulate collagen stability, including chemical crosslinking, thermal or ultraviolet treatment, polymer blending, and mineral addition [12,13,14,15]. Although these methods can enhance degradation resistance, they may also lead to drawbacks such as residual cytotoxicity, altered stiffness, reduced tissue integration, or increased processing complexity [13,16]. Accordingly, there is growing interest in milder modification strategies that achieve controlled stabilization through non-covalent interactions rather than permanent covalent crosslinks.

Amino-acid–mediated modulation represents one such approach. L-serine, a non-essential amino acid containing a polar hydroxyl side chain, participates in hydrogen-bonding interactions and may influence protein hydration [17], molecular packing [18], and supramolecular organization [19]. Compared with other non-covalent modifiers such as sugars, polyols, or bulkier amino acids, L-serine offers a uniquely small, hydroxyl-containing side chain that can participate in hydrogen-bonding and hydration-mediated interactions while introducing minimal steric interference, making it a particularly suitable candidate for subtle modulation of collagen stability. When present within or around protein matrices, hydroxyl groups can promote additional inter- and intramolecular hydrogen bonds, influence hydration shells, and subtly affect fibrillogenesis and fibril packing [20,21]. We hypothesized that incorporating L-serine into collagen matrices could promote additional non-covalent interactions that modestly enhance structural stability and reduce enzymatic accessibility, thereby delaying biodegradation without the need for chemical crosslinkers.

Based on this rationale, the present study focuses on a materials-oriented evaluation of collagen scaffolds supplemented with L-serine, specifically examining whether this modification alters in vivo degradation characteristics. Our aim was not to assess osteogenic or regenerative performance, but rather to determine whether L-serine incorporation can serve as a simple, biocompatible, and non-crosslinking strategy to adjust the resorption profile of collagen-based constructs. For this purpose, we prepared L-serine-containing scaffolds using hydrolyzed bovine skin collagen, a rapidly degradable collagen source commonly used for cell culture studies, which provides a sensitive model for detecting stabilization effects. We then evaluated degradation behavior through structural characterization and subcutaneous implantation. The findings are intended to provide preliminary feasibility data supporting amino-acid–assisted modulation as a gentle stabilization approach for collagen biomaterials. Although osteogenic or regenerative performance is essential for defining the suitability of a biomaterial for tissue engineering, the present study focuses specifically on structural stabilization and degradation behavior. The regenerative capacity of L-serine–modified collagen remains to be established in future biological and defect-specific models. While collagen scaffolds are frequently applied in bone regeneration, the stabilization strategy examined here is not restricted to bone-related uses; rather, the modulation of degradation behavior achieved through L-serine incorporation may be relevant to a wider range of soft- and hard-tissue engineering applications.

## 2. Materials and Methods

### 2.1. Experimental Materials

Type I collagen derived from calf skin (bovine skin; Sigma-Aldrich, Cat. No. C9791, St. Louis, MO, USA) was used as the base material for scaffold fabrication. Lyophilized collagen was dispersed in distilled water to obtain a 2% (*w*/*v*) collagen suspension under gentle stirring at 4 °C. For the experimental groups, L-serine powder (Sigma-Aldrich) was dissolved in distilled water and added to the collagen suspension to yield final concentrations of 10, 20, 30, or 40 wt% L-serine relative to the dry weight of collagen. The selected range of 10–40 wt% L-serine was based on preliminary solubility and formability assessments, which showed that concentrations below 10 wt% produced negligible structural modification, whereas concentrations above 40 wt% led to phase separation and impaired scaffold formation during the drying process. Using distilled water-maintained collagen in a partially hydrated, non-fibrillar state during mixing, which facilitated homogeneous incorporation of L-serine prior to drying.

The mixtures were homogenized to form collagen–L-serine hydrogels, which were then cast into flat polystyrene molds to a uniform thickness of approximately 1 mm and dried at 4 °C for 48 h to produce scaffold sheets. Following drying, the scaffold sheets had a uniform thickness of approximately 1 mm and were trimmed into rectangular specimens measuring 10 × 10 mm for in vitro analyses and 5 × 10 mm for subcutaneous implantation. Bulk density and porosity were not quantified in this study, as the focus was on degradation behavior rather than mechanical or transport properties; these parameters will be addressed in future work. The dried sheets were used for structural analyses and animal experiments. A commercially available bilayer porcine collagen membrane composed of type I and III collagen (Bio-Gide^®^, Geistlich Pharma, Wolhusen, Switzerland) served as a positive control.

### 2.2. X-Ray Diffraction (XRD) Analysis

Lyophilized samples were prepared by freeze-drying the specimens at −80 °C followed by vacuum drying (−50 °C, <0.1 mbar) for 24 h. The dried samples were mounted on a low-background Si holder. XRD patterns were collected using an Ultima IV X-ray diffractometer (Rigaku, Tokyo, Japan) equipped with Cu Kα radiation (λ = 1.5406 Å, 40 kV, 30 mA) in 2θ geometry. Data were acquired over the 2θ range of 5–80° with a step size of 0.02° and a counting time of 0.5 s per step. Phase identification was carried out by comparing diffraction peaks with the International Centre for Diffraction Data Powder Diffraction File (ICDD PDF) database. The peak intensity ratio was calculated by dividing the maximum intensity of a representative crystalline L-serine peak at 2θ = 22–23° by that of the collagen amorphous halo at 2θ = 20° using the digitized XRD profiles. XRD measurements were performed on two independently prepared specimens (*n* = 2).

### 2.3. Fourier Transforms Infrared Spectroscopy (FT-IR) Analysis

FT-IR was employed to identify the chemical structures of the samples, based on their characteristic absorption fingerprints. FT-IR spectra were obtained using an S100 spectrometer (PerkinElmer, Waltham, MA, USA). Lyophilized samples were finely ground and mixed with spectroscopic-grade potassium bromide (KBr) to obtain transparent pellets suitable for transmission analysis, which improves spectral quality by ensuring uniform dispersion and minimizing light scattering. The KBr–sample mixtures were compressed into pellets using a hydraulic press (Specac, Orpington, UK) at 10 tons of pressure for 10 min. FT-IR spectra were obtained using an S100 spectrometer (PerkinElmer, Waltham, MA, USA) at a resolution of 4 cm^−1^, recorded over the range of 450–4000 cm^−1^, with each spectrum representing the average of 23 scans. FT-IR analysis was conducted on two independent samples (*n* = 2).

### 2.4. Circular Dichroism (CD) Spectrometer Analysis

CD spectroscopy was performed to analyze the secondary structure of the samples using a spectropolarimeter (Jasco, Easton, MD, USA). Samples were placed in a 10-mm path-length quartz high-precision cuvette (Jasco, Easton, MD, USA). Spectra were recorded from 190 to 260 nm, with each measurement averaged over three scans. Prior to measurement, baseline correction was performed using distilled water. Data were expressed as ellipticity (mdeg) as a function of wavelength. Spectra were smoothed using the Savitzky–Golay filter in the Spectra Analysis software (version 2.11.01, Jasco). CD spectra were obtained from two independently prepared samples (*n* = 2).

### 2.5. Scanning Electron Microscopy (SEM) Analysis

The surface morphology of Biogide^®^, collagen, and collagen + L-serine specimens was examined by scanning electron microscopy (JSM-IT200, JEOL, Tokyo, Japan). For sample preparation, the hydrogel form of collagen + L-serine was fixed in 2.5% glutaraldehyde for 2 h at 4 °C, followed by sequential dehydration in graded ethanol solutions (30%, 50%, 70%, 90%, and 100%). After dehydration, the samples were slowly dried at room temperature to minimize structural collapse and then sputter-coated with a thin platinum layer. SEM images were obtained at magnifications of ×500 and ×5000 under an accelerating voltage of 10 kV, providing detailed views of fibrillar organization and surface topography. SEM imaging was performed using two independent samples (*n* = 2), with representative micrographs collected from multiple regions of each specimen.

### 2.6. Animals’ Studies

This study was approved by the Institutional Animal Care and Use Committee of Gangneung-Wonju National University (GWNU-2025-12). Twenty-four male Institute of Cancer Research (ICR) mice (7 weeks old, 33–39.5 g) were obtained from Samtako Bio Inc. (Osan, Republic of Korea). Animals were housed two per cage and allowed a one-week acclimation period before experimentation. Environmental conditions were maintained at 20–22 °C under a 12 h light/dark cycle, with free access to food and water.

Mice were randomly assigned to three groups (*n* = 8 per group) according to the implanted material: (1) a commercial collagen membrane (Bio-Gide^®^), (2) unmodified type I collagen (Sigma-Aldrich, St. Louis, MO, USA), or (3) collagen containing 40 wt% L-serine. The sample size (*n* = 8) was chosen in accordance with common practice for exploratory subcutaneous degradation models and was not based on a formal a priori power calculation, as the aim was to obtain preliminary biological data on scaffold persistence rather than to test a predefined statistical hypothesis. To minimize confounding factors, mice within the same cage were assigned to the same group, and cages from different groups were placed adjacently to control for cage-location effects. Group allocation was concealed from all researchers except the corresponding author, and investigators performing the experiments and analyses were blinded to the group assignments.

Mice were anesthetized by intramuscular injection of Zoletil (Virbac Korea, Seoul, Republic of Korea) and Rompun (Bayer Korea, Seoul, Republic of Korea). A dorsal skin incision was made, the subcutaneous tissue was dissected, and the prepared material (20 mg) was implanted subcutaneously. The incision was closed with sutures. To prevent infection, gentamicin (5 mg/kg; Samu Median, Seoul, Republic of Korea) and tolfenamic acid (0.1 mL/kg; Samyang Anipharm, Seoul, Republic of Korea) were administered intramuscularly immediately after surgery and again on postoperative day 1.

Sonographic imaging was performed using an ultrasound system (ACCUVIX V10^®^, Samsung Medison, Seoul, Republic of Korea) to evaluate the residual graft in the subcutaneous pocket. Sonographic imaging of the graft site was performed under anesthesia (Zoletil, intramuscular; or sevoflurane, inhalation) at the following time points: before implantation, immediately after surgery, and at 1 and 3 weeks postoperatively. At 3 weeks, mice were euthanized by CO_2_ inhalation, and the graft sites, including the underlying muscle tissue, were harvested for histological analysis. An implantation duration of three weeks was selected based on preliminary pilot observations indicating substantial degradation of collagen scaffolds within this period, which allowed clear comparison among groups. This timeframe is also consistent with previous short-term subcutaneous biodegradation studies of collagen-based materials.

Tissue specimens were fixed in 10% neutral-buffered formalin, paraffin-embedded, and sectioned at 5 µm. Picrosirius Red staining was performed for collagen visualization. Sections were deparaffinized, rehydrated, stained with Picro Sirius Red (Abcam, Cambridge, UK), rinsed with acetic acid, dehydrated, and mounted. Images were captured using a DP72 digital camera (Olympus, Tokyo, Japan).

Quantification of residual graft area was performed exclusively on these histological sections. Digital images were analyzed using SigmaScan Pro 5.0 (SPSS Inc., Chicago, IL, USA). The residual graft was identified based on its dense, eosinophilic collagen appearance. Using the manual tracing tool, the boundary of the remaining graft material was outlined, and the software automatically calculated the enclosed area in square millimeters. Three non-overlapping sections were measured per sample, and the mean value was used for statistical comparison.

### 2.7. Statistical Analysis

Quantitative data from sonographic imaging (residual graft area over time) and histological evaluation (percentage of residual material and collagen deposition) were expressed as mean ± standard deviation (SD). Normality of data distribution was tested using the Shapiro–Wilk test. Homogeneity of variance was assessed using Levene’s test. Comparisons among groups were performed using one-way analysis of variance (ANOVA), followed by Tukey’s post hoc test for multiple comparisons. A *p*-value < 0.05 was considered statistically significant. Statistical analyses were conducted using GraphPad Prism version 10.0 (GraphPad Software, San Diego, CA, USA).

## 3. Results

### 3.1. Structural and Morphological Characterization

**Figure 1:**
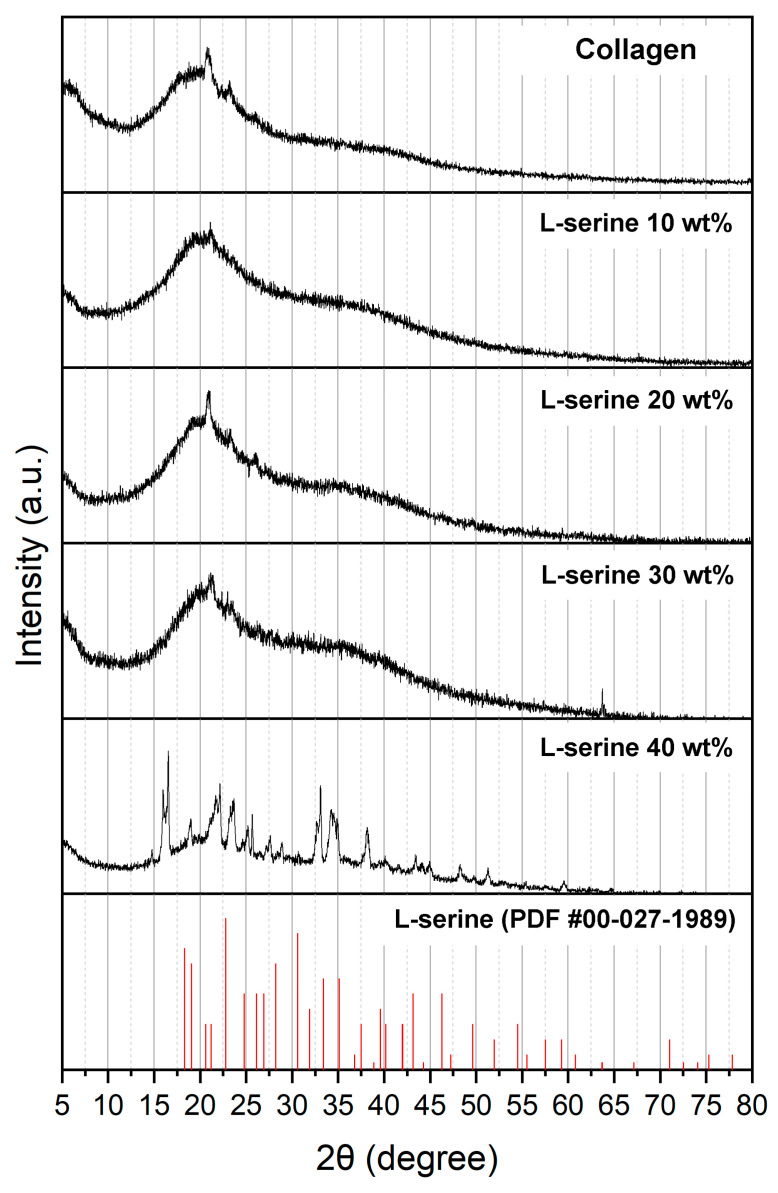
XRD patterns of pure collagen and collagen-L-serine composites with different L-serine contents (10, 20, 30, and 40 wt%), together with the reference diffraction pattern of crystalline L-serine (red sticks, PDF #00-027-1989). Broad amorphous halos dominate the patterns up to 30 wt% L-serine, whereas multiple sharp crystalline peaks appear at 40 wt%. The peak intensity ratio between the crystalline L-serine peak (2θ = 22–23°) and the collagen amorphous halo (2θ = 20°) increased from 0.85–0.94 (10–30 wt%) to 1.12 at 40 wt%, indicating a clear compositional phase transition.

The phase evolution of collagen–L-serine composites was investigated by XRD (Figure 1). Pure collagen showed a broad amorphous halo with a main reflection near 20°, characteristic of the collagen matrix. No distinct crystalline reflections were observed, confirming the predominantly amorphous nature of the material. With the incorporation of L-serine up to 30 wt%, the diffraction profiles remained like that of pure collagen, displaying broad amorphous peaks without pronounced sharp reflections. This indicates that L-serine was largely dispersed or molecularly associated within the collagen network and did not form its own crystalline domains at these concentrations. In contrast, the composite containing 40 wt% L-serine exhibited multiple sharp reflections in the range of 15–30° and beyond, which matched well with those of the reference L-serine pattern (PDF #00-027-1989), confirming the presence of free L-serine crystals. These findings indicate that a compositional threshold exists between 30 wt% and 40 wt% L-serine. Below this range, L-serine remains predominantly amorphous within the collagen matrix, whereas at 40 wt% it precipitates as independent crystalline domains. This pronounced increase in crystallinity at higher L-serine loading is expected to influence the composite’s mechanical integrity and degradation behavior as discussed in the following sections. Overall, the XRD results indicate a dose-dependent effect of L-serine on the structural organization of collagen. At low-to-moderate concentrations (10–30 wt%), serine appeared to promote partial ordering within the collagen matrix without disrupting the amorphous background, whereas at high concentration (40 wt%), crystalline L-serine dominated the diffraction pattern, suggesting the onset of independent serine crystallization within the composite. To further support the compositional transition observed in the XRD patterns, a semi-quantitative comparison was performed using the intensity ratio between a representative crystalline peak of L-serine at 2θ = 22–23° and the amorphous collagen halo at 2θ = 20°. The calculated peak ratios were 0.85, 0.87, 0.94, and 1.12 for the 10, 20, 30, and 40 wt% L-serine groups, respectively. The values for 10–30 wt% remained within a relatively narrow range (within approximately 10% variation), indicating only minor changes in the contribution of crystalline L-serine in this compositional interval. In contrast, the ratio increased by more than 30% at 40 wt% compared with the 10 wt% group, confirming a marked enhancement of the crystalline L-serine phase at this higher loading.

**Figure 2:**
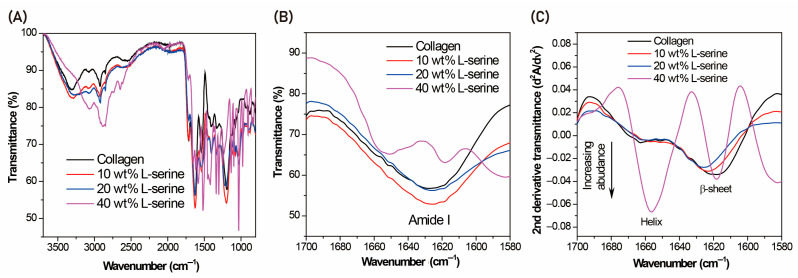
FT-IR spectra of collagen with increasing L-serine incorporation, shown in three panels: (**A**) full-range FT-IR (3500–1000 cm^−1^) displaying the broad OH/NH stretching band (~3200–3400 cm^−1^) which intensifies with higher serine content, along with characteristic amide I (~1656 cm^−1^) and amide II (~1550 cm^−1^) bands; (**B**) expanded amide I region (1600–1700 cm^−1^) highlighting a decrease in the collagen triple-helix peak at ~1656 cm^−1^ and the concurrent growth of a band at ~1632 cm^−1^ indicative of β-sheet structures as L-serine content increases; (**C**) second-derivative spectra of the amide I region, resolving overlapping components and confirming the serine-induced structural changes—namely, the pronounced emergence of the β-sheet-associated ~1632 cm^−1^ peak and a reduction of the triple-helix ~1656 cm^−1^ band with greater L-serine incorporation.

The full-range FT-IR spectra (3500–1000 cm^−1^) of collagen with added L-serine show a broad O–H/N–H stretching band (~3200–3400 cm^−1^) that intensifies progressively as serine content increases (Figure 2A). The characteristic amide I (~1656 cm^−1^) and amide II (~1550 cm^−1^) bands of collagen are present in all samples, although their relative intensities shift upon serine incorporation (Figure 2A). Focusing on the amide I region (1600–1700 cm^−1^) reveals clear serine-induced secondary structure changes: the dominant triple-helix peak at ~1656 cm^−1^ diminishes in intensity with higher serine levels (e.g., 20–40% by weight) (Figure 2B). Concomitantly, a distinct new band emerges around ~1632 cm^−1^ and grows more pronounced, indicating the formation of β-sheet structures as L-serine content rises (Figure 2B). To resolve overlapping amide I components, second-derivative spectra of the amide I region were analyzed rather than performing full curve-fitting deconvolution (Figure 2C). The second-derivative analysis confirms these structural alterations, showing a well-defined β-sheet–associated component at ~1632 cm^−1^ that becomes increasingly prominent with greater L-serine, alongside a corresponding reduction of the triple-helix-associated ~1656 cm^−1^ component in high-serine samples (Figure 2C). These assignments are consistent with widely reported amide I band positions, where triple-helical collagen typically exhibits a maximum near 1650–1660 cm^−1^, while β-sheet–rich structures give rise to bands in the 1625–1640 cm^−1^ range. Accordingly, the present FT-IR analysis provides a qualitative/semi-quantitative indication of secondary structural redistribution rather than a fully deconvoluted quantitative fit.

**Figure 3:**
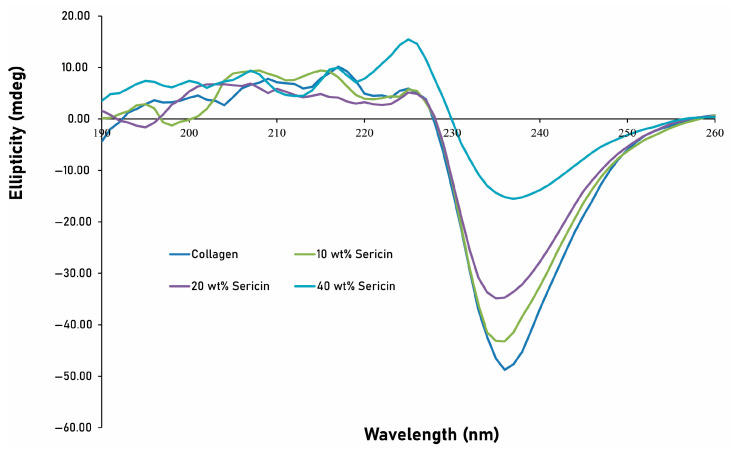
CD spectra of collagen and L-serine–incorporated collagen composites. Native collagen exhibited a strong negative band near 195 nm and only a minimal positive feature around 220–230 nm, consistent with low triple-helical content under the measurement conditions. Incorporation of 10 wt% and 20 wt% L-serine caused only slight changes in ellipticity, indicating minimal perturbation of the spectral profile. In contrast, the 40 wt% L-serine specimens showed an increased positive band at ~224 nm and a reduced negative band intensity, reflecting serine-induced reorganization of the collagen matrix. These spectral changes indicate altered supramolecular arrangement rather than restoration of native triple-helical structure and are consistent with the secondary-structure perturbations identified by FT-IR and the aggregation-associated features observed in XRD.

CD spectra provided additional insight into the conformational changes induced by L-serine incorporation (Figure 3). Native collagen exhibited a strong negative band at ~195 nm (−0.97 mdeg), with only a minimal positive feature near 221 nm, reflecting low triple-helical content under these experimental conditions. Incorporation of 10 wt% L-serine yielded a weak positive band at 219 nm (+0.06 mdeg), while 20 wt% produced a similarly small signal (+0.05 mdeg at 223 nm), indicating only minor perturbation of the overall spectral profile. In contrast, the 40 wt% L-serine specimen displayed a more prominent positive band at 224 nm (+0.20 mdeg) accompanied by a reduced negative band intensity (−0.54 mdeg). Rather than indicating restoration of native triple-helical structure, this shift is more consistent with L-serine-induced alterations in supramolecular organization, including aggregation, hydration changes, or serine-associated ordering effects.

Accordingly, the CD data suggest that high L-serine content modifies the long-range optical activity of the collagen network but does not contradict the FT-IR evidence of local secondary-structure disruption. Together, the findings indicate that 40 wt% L-serine produces significant reorganization of the collagen matrix rather than true enhancement of triple-helical integrity.

**Figure 4:**
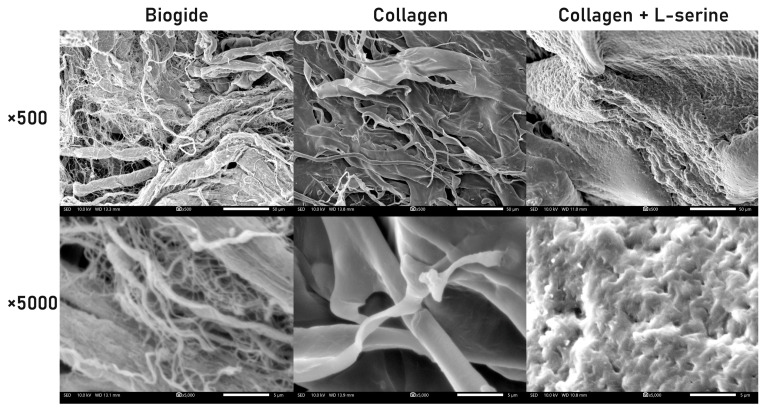
SEM images of Biogide^®^, collagen, and collagen incorporated with 40 wt% L-serine at different magnifications. Representative SEM micrographs at ×500 (top row) and ×5000 (bottom row) show distinct surface morphologies. Biogide^®^ exhibited a porous fibrous architecture with interwoven collagen bundles. The collagen group displayed sheet-like lamellar structures with relatively smooth surfaces. In contrast, collagen incorporated with L-serine demonstrated a compact, irregular, and roughened surface texture. At higher magnification (×5000), Biogide^®^ maintained fibrillar networks, collagen showed flattened ribbon-like fibrils, and collagen + L-serine revealed a dense, granular morphology, suggesting structural modification and altered porosity following L-serine incorporation. Scale bars = 50 μm (×500) and 5 μm (×5000).

SEM analysis was conducted to directly visualize the morphological differences among Biogide^®^, unmodified collagen, and collagen incorporated with 40 wt% L-serine (Figure 4). At low magnification (×500), Biogide^®^ displayed a porous, fibrous network with interwoven collagen bundles, whereas unmodified collagen appeared as relatively flat, sheet-like lamellae with smoother surfaces. In contrast, collagen + L-serine exhibited a denser, compact surface with irregular topography. At higher magnification (×5000), these distinctions became more apparent: Biogide^®^ preserved well-defined fibrillar networks with interconnected pores, unmodified collagen showed flattened ribbon-like fibrils with limited surface roughness, and the L-serine–incorporated collagen presented a granular, roughened morphology suggestive of altered fibril packing. These morphological modifications provide structural context for the reduced porosity and increased resistance to enzymatic degradation observed in the in vivo experiments.

### 3.2. In Vivo Degradation and Histological Evaluation

**Figure 5:**
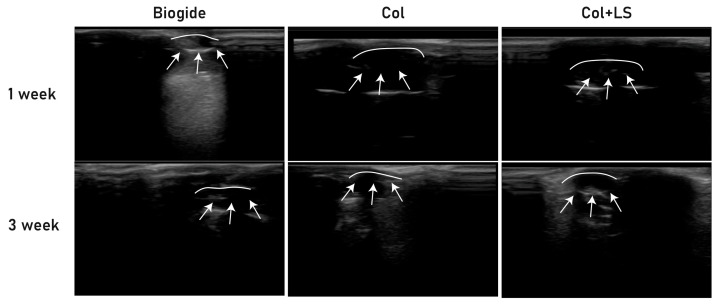
Sonographic evaluation of graft degradation in dorsal skin of mice at 1 and 3 weeks post-implantation. Biogide^®^ (**left**) appeared as a dense echogenic area that persisted at 3 weeks, indicating slower degradation. Collagen (**middle**) showed substantial reduction in echogenicity over time, reflecting rapid biodegradation. Collagen + L-serine (**right**) maintained clearer echogenic boundaries compared with collagen alone, suggesting enhanced stability. White arrows indicate the margins of the implanted graft material.

Serial sonographic imaging demonstrated distinct degradation patterns among the three graft materials (Figure 5). At 1 week, all grafts were clearly visible beneath the dorsal skin, with the Biogide^®^ membrane presenting as a dense, highly echogenic area (i.e., displaying strong ultrasound reflection and appearing bright on the image), while both unmodified collagen and collagen with L-serine (Col + LS) appeared as less dense echogenic structures. By 3 weeks, Biogide^®^ remained partially detectable with reduced but persistent echogenicity, indicating slower degradation. In contrast, unmodified collagen showed marked reduction in echogenic area, consistent with rapid biodegradation. Collagen containing 40 wt% L-serine retained a more discernible echogenic structure than unmodified collagen, suggesting that L-serine incorporation slowed graft absorption and improved stability in vivo.

**Figure 6:**
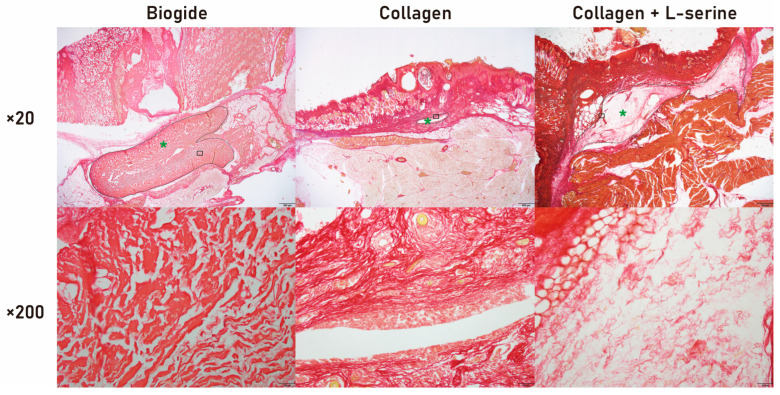
Picrosirius Red staining of implanted grafts in vivo. Representative histological images of the grafted sites at 3 weeks post-implantation in the Bio-Gide^®^, collagen, and collagen + L-serine groups. Low-magnification views (×20, **top row**) show the overall graft morphology, while high-magnification views (×200, **bottom row**) correspond to the boxed regions in the upper panels. The residual graft area is indicated by a green asterisk (*), and the graft boundaries are delineated with a black line. Collagen fibers are visualized as red-stained structures. Scale bars: 500 µm (×20) and 50 µm (×200).

Histological evaluation using Picro-Sirius Red (PSR) staining at 3 weeks revealed distinct patterns of collagen persistence among the experimental groups (Figure 6). In the Bio-Gide^®^ group, a clearly defined residual collagen structure was visible at low magnification (×20), exhibiting a dense, continuous red-stained matrix with well-preserved shape and uniform collagen distribution. At higher magnification (×200), thick collagen bundles remained intact and closely packed, indicating relatively slow degradation within the implantation site. In the collagen-only group, PSR-positive material was sparsely distributed and primarily fragmented, with weak staining intensity at both magnifications. Residual collagen appeared discontinuous and scattered, suggesting rapid degradation and limited remaining scaffold structure. In the collagen + L-serine group, residual collagen was still present but demonstrated an intermediate pattern between the two other groups. At low magnification, the material showed partially recognizable structure but with less uniformity and border clarity compared to Bio-Gide^®^. At higher magnification, collagen bundles appeared thinner, loosely packed, and more irregular, indicating partial preservation with ongoing resorption. Overall, the PSR-stained results qualitatively supported the trend observed in the degradation analysis: Bio-Gide^®^ showed the greatest structural persistence, collagen-only degraded most rapidly, and the collagen + L-serine group demonstrated intermediate collagen retention.

Supplementary histological evaluation using H&E staining demonstrated clear qualitative differences in residual scaffold morphology and tissue response among treatment groups (Figure S1). In the Bio-Gide^®^ group, a well-defined and continuous residual membrane-like structure remained visible, with dense eosinophilic material and minimal inflammatory cell infiltration, consistent with a clearly defined residual boundary was observed, and high-magnification views showed densely packed eosinophilic collagen bundles with notable infiltration of inflammatory cells, suggesting active remodeling and degradation mediated by host immune responses. In the collagen-only group, only small, faint, and irregular eosinophilic remnants were present within a poorly defined residual zone, consistent with rapid degradation and loss of scaffold continuity. The collagen + L-serine group exhibited an intermediate pattern, with a partially preserved scaffold-like region showing thinner and more fragmented eosinophilic fibers. In the high-magnification field examined for this group, inflammatory cell infiltration appeared limited, indicating comparatively less active cellular degradation at that specific site. Together, these H&E observations illustrate differences in residual material morphology and the degree of inflammatory involvement among the implanted materials, in line with the overall degradation trends observed in PSR-stained sections.

**Figure 7:**
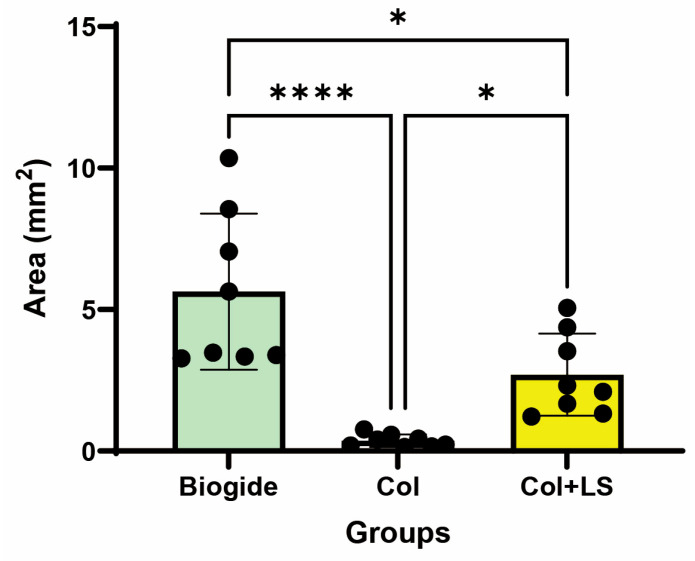
Quantitative analysis of residual graft area at 3 weeks post-implantation. Bar graphs show mean ± SD with individual data points (*n* = 8 per group). Biogide^®^ exhibited the largest residual graft area, collagen + L-serine showed intermediate persistence, and collagen-only degraded most rapidly. Statistical analysis by one-way ANOVA with Tukey’s multiple comparisons demonstrated significant differences among all groups (* *p* < 0.05; **** *p* < 0.0001).

Quantitative histomorphometric analysis at 3 weeks confirmed significant differences in graft persistence among the three groups (Figure 7). The residual graft area was largest in the Biogide^®^ group (mean ± SD: 5.64 ± 2.76 mm^2^), followed by the collagen + L-serine group (2.70 ± 1.45 mm^2^), and smallest in the collagen-only group (0.37 ± 0.22 mm^2^). One-way ANOVA with Tukey’s post hoc test revealed that Biogide^®^ had significantly greater residual area than both collagen-only (*p* < 0.0001) and collagen + L-serine (*p* = 0.0102). In addition, collagen + L-serine retained significantly more residual material than collagen-only (*p* = 0.0438). These findings indicate that L-serine incorporation slowed the biodegradation of collagen compared with unmodified collagen, although Biogide^®^ remained the most stable material.

## 4. Discussion

In this study, we demonstrated that incorporation of L-serine into collagen matrices modulated their structural organization and in vivo degradation profile without the use of chemical crosslinking. Structural analyses revealed that low-to-moderate concentrations of L-serine (10–30 wt%) preserved the triple-helical architecture of collagen while modestly increasing β-sheet content, whereas higher incorporation (40 wt%) resulted in the appearance of crystalline serine-associated domains and redistribution toward β-sheet enrichment (Figure 1, Figure 2 and Figure 3). In vivo experiments further showed that collagen containing 40 wt% L-serine exhibited slower resorption compared with unmodified collagen, although its persistence remained lower than that of the commercially processed collagen membrane, Bio-Gide^®^ (Figure 6 and Figure 7). Collectively, these findings support the feasibility of amino acid–based modulation as a gentle and non-crosslinking approach for controlling collagen biodegradation.

Traditional strategies for prolonging collagen stability often rely on chemical crosslinkers or physical treatments [11,12,13,14]. While agents such as glutaraldehyde and genipin can substantively reduce enzymatic susceptibility, their use has been associated with residual cytotoxicity risks or compromised tissue integration [16,22]. Non-chemical approaches, such as dehydrothermal and ultraviolet treatments, may extend stability but can also alter mechanical behavior and reduce handling flexibility [12,13]. Polymer blending and mineral reinforcement can enhance biostability, yet these techniques may introduce fabrication complexity and reduce clinical adaptability [13,15]. In contrast, L-serine incorporation represents a simple, aqueous, and biocompatible modification approach that avoids harsh reagents while maintaining key intrinsic characteristics of collagen.

**Figure 8:**
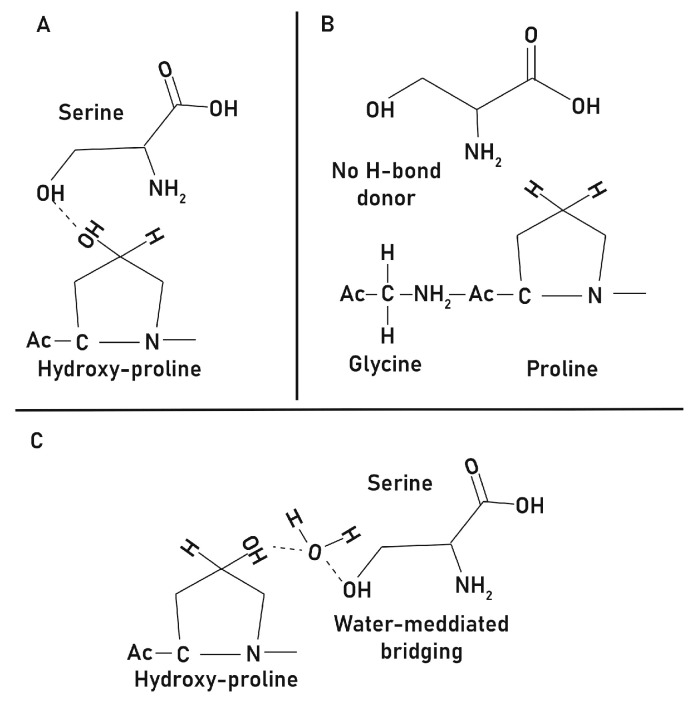
Proposed hydrogen bonding mechanisms between serine and collagen components. (**A**) Direct hydrogen bonding between free serine and hydroxyproline, where the hydroxyl group of serine interacts with the hydroxyl group of hydroxyproline. (**B**) Glycine–proline pairing lacks a hydrogen bond donor, illustrating the absence of stabilizing interaction compared with hydroxyproline–serine. (**C**) Water-mediated ternary network bridging, in which hydroxyproline and serine interact indirectly through hydrogen bonds with a bridging water molecule. Together, these interactions suggest that serine can stabilize collagen matrices by forming direct or water-mediated hydrogen bond networks.

The stabilizing effect observed in this work is likely related to the hydroxyl-containing side chain of L-serine, which can participate in hydrogen bonding with collagen backbones and influence hydration-related interactions (Figure 8). Moderate incorporation levels (10–30 wt%) may enhance non-covalent intermolecular packing, thereby reducing enzymatic accessibility to cleavage sites [23,24]. In hydrated protein matrices, hydroxyl-containing amino acids can promote both direct hydrogen bonding and water-mediated bridging interactions, leading to tighter intermolecular packing and the formation of a more structured hydration shell [17,18,19,20]. Such ‘bound’ or ordered water has reduced mobility compared with bulk water and has been associated with decreased susceptibility of collagenous substrates to enzymatic degradation, because proteolytic enzymes require both physical access and local backbone flexibility to cleave peptide bonds [23,24]. Consistent with this interpretation, FT-IR and CD analyses suggested partial preservation of triple-helical features under moderate doping conditions (Figure 2 and Figure 3).

In contrast, at 40 wt%, the emergence of crystalline domains and increased β-sheet signatures suggested that excessive serine content could disrupt helicity and promote aggregation (Figure 1 and Figure 4). These changes suggest a transition from subtle hydrogen-bond–mediated modulation to more extensive supramolecular reorganization, in which aggregation and phase separation alter packing density and generate heterogeneous regions within the scaffold. Although the 40 wt% formulation exhibited relatively slower in vivo degradation, this outcome is more plausibly explained by altered supramolecular organization and uneven enzymatic accessibility rather than preservation of triple-helical integrity. The formation of serine-rich crystalline regions and β-sheet–rich aggregates disrupt the continuity of the collagen network and modifies hydration and packing characteristics, producing effects analogous to—but ultimately distinct from—the hydrogen-bond–mediated stabilization reported for polyol-based additives [21,25]. At higher loadings, therefore, the balance shifts from beneficial hydrogen-bond interactions to structural alteration and aggregation, which limit any potential stabilizing effect on the collagen triple helix. It should be noted that the hydrogen-bonding and hydration-mediated explanations proposed here are mechanistic hypotheses based on indirect spectroscopic and degradation trends rather than direct measurements. This study did not quantify changes in hydration dynamics, L-serine–collagen binding interactions, or protease accessibility, which would be required to definitively establish the mechanism. Accordingly, the interpretations presented here should be viewed as plausible but provisional, and future work incorporating hydration analysis, binding assays, and controlled collagenase degradation studies will be necessary to validate and refine these mechanistic insights.

The subcutaneous implantation model provided an initial in vivo evaluation of degradation behavior (Figure 5). As expected, unmodified collagen degraded rapidly, consistent with its well-known enzymatic susceptibility [26,27]. Collagen containing 40 wt% L-serine showed significantly greater residual area after 3 weeks (Figure 6 and Figure 7), supporting the hypothesis that L-serine incorporation can delay proteolysis under physiological conditions. Bio-Gide^®^ remained the most stable material, reflecting performance optimization through industrial processing. While previous studies have reported biological activity of L-serine, including osteoblast-supportive and osteoclast-modulating properties [28,29], such cellular responses were not evaluated in the present work, and therefore no regenerative or osteogenic conclusions can be drawn based on the current data. The applicability of the L-serine–modified collagen scaffolds is not limited to bone repair. Collagen is broadly used across soft-tissue augmentation, periodontal regeneration, wound healing, and guided tissue regeneration, where controlled degradation and structural stability are important design parameters [30]. Because this study evaluated physicochemical stabilization and in vivo persistence rather than bone-specific biological responses, further investigations—including osteogenic assays, soft-tissue interaction studies, and defect-specific in vivo models—will be required to determine the optimal clinical indication for these formulations.

This study has several strengths, including the use of complementary structural characterization methods and quantitative in vivo degradation analysis. Nonetheless, important limitations must be acknowledged. First, the implantation duration was limited to 3 weeks, which limits interpretation regarding long-term persistence. Second, only a single L-serine concentration was evaluated in vivo, preventing comprehensive assessment of dose–response relationships. Third, mechanical properties relevant to clinical handling, including tensile strength, elasticity, and suture pullout resistance, were not examined. Another limitation is the absence of In Vitro degradation assays using collagenase solutions, which would allow a clearer mechanistic understanding of collagen susceptibility under controlled enzymatic conditions. Incorporating collagenase-based assays in future studies will help correlate structural modifications with enzyme-mediated degradation kinetics. While the present work demonstrates that L-serine incorporation can modulate collagen structure and slow in vivo degradation at 40 wt%, the regenerative or osteogenic performance of the modified scaffolds was not evaluated. Such properties are essential for tissue-engineering applications. Therefore, additional investigations—including cell-based biocompatibility and differentiation assays, evaluation of host–material interactions, and assessment in defect-specific bone or soft-tissue models—will be required to determine whether L-serine–enriched collagen scaffolds meet the functional requirements for regenerative use. Addressing these limitations will be essential for future development. Expanded studies should incorporate extended implantation timelines, include additional concentrations of L-serine, and conduct mechanical testing. Moreover, evaluation in anatomically relevant models, such as bone-contact or defect-based environments, would provide more meaningful context for potential application-specific performance [31,32].

## 5. Conclusions

This study demonstrates that incorporating L-serine into collagen matrices offers a simple, non-crosslinking means of modulating scaffold degradation through concentration-dependent alterations in supramolecular organization. Unlike conventional stabilization methods involving chemical crosslinkers, polyol additives, or mineral reinforcement, the use of a naturally occurring amino acid enables structural tuning without introducing reactive residues or substantially changing processing conditions. The findings highlight a conceptual advance in which degradation behavior can be adjusted through mild, hydration- and packing-mediated interactions rather than through covalent modification. While further work is needed to assess mechanical performance, enzymatic stability, and biological outcomes in defect-specific models, this amino-acid–assisted strategy provides a promising foundation for developing collagen-based biomaterials where gentle, biocompatible control of resorption is desirable.

## Data Availability

The original contributions presented in the study are included in the article, further inquiries can be directed to the corresponding author.

## Supplementary Materials

The following supporting information can be downloaded at: https://www.mdpi.com/article/10.3390/jfb16120466/s1, Figure S1: Comparative H&E Morphology of Bio-Gide^®^, Collagen-Only, and L-Serine–Incorporated Collagen at 3 Weeks.

## Abbreviations

The following abbreviations are used in this manuscript:

ANOVA	Analysis of Variance
CD	Circular Dichroism
FT-IR	Fourier-Transform Infrared Spectroscopy
H&E	Hematoxylin and Eosin
ICDD PDF	International Centre for Diffraction Data—Powder Diffraction File
PBS	Phosphate-Buffered Saline
PSR	Picrosirius Red
SD	Standard Deviation
SEM	Scanning Electron Microscopy
XRD	X-ray Diffraction
wt%	Weight Percent
