# Supplementary material for: L-Serine–Incorporated Collagen Scaffolds for Modulating In Vivo Degradation Behavior
Source: J Funct Biomater. 2025 Dec 18;16(12):466. doi: 10.3390/jfb16120466 (PMC12733836; doi:10.3390/jfb16120466)

Figure S1: Comparative H&E Morphology of Bio-Gide®, Collagen-Only, and L-Serine–Incorporated Collagen at 3 Weeks. (A–C) Low-magnification H&E-stained sections showing residual graft areas outlined with an irregular black line. (A) Bio-Gide® group demonstrating a well-preserved membrane structure with a clearly defined residual boundary. (B) Collagen-only group showing minimal remaining eosinophilic material and a poorly defined residual zone, consistent with rapid degradation. (C) Collagen + L-serine group displaying a partially preserved scaffold region with intermediate continuity. Small rectangles in A and C indicate the locations selected for high-magnification imaging. (D–E) High-magnification views corresponding to the boxed regions in panels A and C. (D) Bio-Gide® shows densely packed eosinophilic collagen bundles with notable infiltration of inflammatory cells, suggesting active remodeling and degradation mediated by host immune responses. (E) Collagen + L-serine demonstrates thinner and more fragmented eosinophilic fibers. This morphology is consistent with the use of hydrolyzed collagen and shows limited visible inflammatory infiltration in this field, indicating comparatively less active cellular degradation at the examined site. Together, these H&E observations illustrate differences in residual material morphology and inflammatory involvement among the implanted materials, corresponding to the overall degradation trend observed in PSR-stained sections.

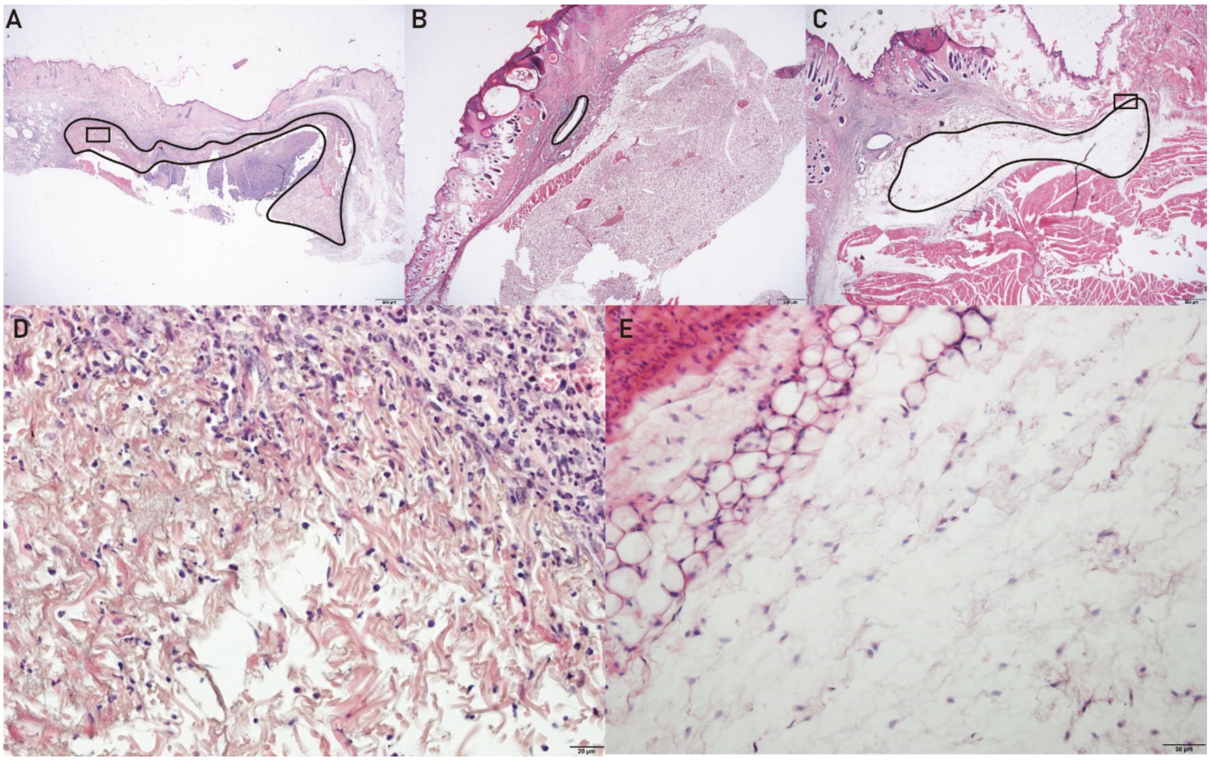

Supplement: Supplementary file 1 [file jfb-16-00466-s001.zip › jfb-4024542-supplementary.pdf]
